# Effect of Bioactive Glass into Mineral Trioxide Aggregate on the Biocompatibility and Mineralization Potential of Dental Pulp Stem Cells

**DOI:** 10.34133/bmr.0142

**Published:** 2025-02-07

**Authors:** Hee-Gyun Kim, Bin-Na Lee, Hyun-Jeong Jeong, Hyun-Jung Kim, Jiyoung Kwon, Soram Oh, Duck-Su Kim, Kyoung-Kyu Choi, Reuben H. Kim, Ji-Hyun Jang

**Affiliations:** ^1^ Department of Conservative Dentistry, Graduate School, Kyung Hee University, Seoul, Korea.; ^2^ Department of Conservative Dentistry, School of Dentistry, Dental Science Research Institute, Chonnam National University, Gwangju, Korea.; ^3^ Department of Conservative Dentistry, School of Dentistry, Kyung Hee University, Seoul, Korea.; ^4^ Section of Restorative Dentistry, School of Dentistry, University of California Los Angeles, Los Angeles, CA, USA.

## Abstract

**Introduction:** Previous studies have shown that bioactive glass (BG) can enhance the formation of hydroxyapatite under simulated body fluid (SBF) conditions when combined with mineral trioxide aggregate (MTA). This study aims to assess the impact of BG-supplemented MTA on the biocompatibility and mineralization potential of dental pulp stem cells (DPSCs). **Methods:** We prepared ProRoot MTA (MTA) and MTA supplemented with 2% and 4% BG. Five passages of DPSCs were utilized for the experiments. The DPSCs were subjected to various tests to determine their morphology, viability, cell migration, and adhesion assay. Additionally, mineralization ability was assessed through SBF immersion treatment, alkaline phosphatase (ALP) activity test, Alizarin red S (ARS) staining, and real-time quantitative polymerase chain reaction (RT-qPCR) analysis. **Results:** The biocompatibility of BG-supplemented MTA was found to be comparable to that of conventional MTA, as demonstrated by the cell counting kit-8 (CCK-8) assay, cell migration, adhesion assays, and cell morphology on cement surfaces. Under SBF treatment, MTA supplemented with BG, particularly at a concentration of 4%, exhibited higher mineralization potential than conventional MTA in the ALP activity assay. This was supported by denser ARS staining, increased ALP activity, and higher expression of dentin sialophosphoprotein (DSPP), ALP, and bone morphogenetic protein-2 (BMP-2) in the SBF-treated MTABG group. **Conclusion:** Our study revealed that the biocompatibility of BG-supplemented MTA is similar to that of conventional MTA. Additionally, under SBF treatment, BG-supplemented MTA displayed enhanced mineralization potential, indicating that BG supplementation can augment the mineralization capabilities of MTA.

## Introduction

Calcium silicate-based cement has become a crucial material in endodontic therapy since the introduction of mineral trioxide aggregate (MTA) in 1993 [1]. It possesses superior properties, such as biocompatibility, bioactivity, and sealing ability [2], and has revolutionized various endodontic procedures, including pulp capping, root-end filling, pulpotomy, apexification, and perforation repair [3]. Additionally, MTA has been found to be less toxic and biocompatible, promoting the proliferation and differentiation of dental pulp stem cells (DPSCs) when used as a pulp-capping material [4].

However, MTA has certain shortcomings reported in several studies [5–7], including a long setting time [8], inconvenient operability [5], and poor physical properties in the presence of blood before the final setting [6]. To overcome these disadvantages, additives like calcium chloride [7] and methylcellulose have been used to decrease setting times and improve handling properties.

Bioactive glass (BG) was first introduced in 1969 as a stable and nearly inert bone implant material [9]. It has been found to enhance osteogenesis in hard tissue defect areas through the reaction with body fluid, leading to the regeneration of tissues, a process known as osteostimulation [9]. Recent studies have utilized BG as an additive to enhance the remineralization ability of dental restoration materials [10–14]. Previous research has shown that BG-incorporated MTA increases dentin push-out bond and compressive strengths under simulated body fluid (SBF) storage conditions without disrupting the MTA maturation reaction [11].

This study aims to evaluate the effects of BG-supplemented MTA on the biocompatibility of DPSCs through cell viability, migration, and adhesion assays. Additionally, the study will investigate the effects of BG-supplemented MTA on the mineralization potential of DPSCs with or without immersion in SBF solution.

## Materials and Methods

### Material preparation

Primary cultures of human DPSCs were obtained from the company Cefobio in Seoul, Korea. These stem cells were isolated from premolars and supernumerary teeth, with donors ranging in age from 6 to 20 years. The cells were pooled together and cultured. Flow cytometry analysis confirmed that the cells were positive for CD105 (Endoglin) and STRO-1, with less than 10% positivity for the latter. Additionally, polymerase chain reaction (PCR) screening showed that Nestin 1 was also positive in these cultures.

For specimen preparation, DW was used as the liquid solution in all experimental groups. The group names were named based on the type of powder used to prepare the MTA specimens. White ProRoot MTA (Dentsply Sirona, Tulsa, USA) served as the control group and is labeled as MTA. The primary components of MTA powder were tricalcium silicate (Ca_3_SiO_5_), dicalcium silicate (Ca_2_SiO_4_), tricalcium aluminate (Ca_3_Al_2_O_6_), bismuth oxide (Bi_2_O_3_), and calcium sulfate (CaSO_4_). The MTA with BG-supplemented groups (BG/MTA groups) were prepared using the MTA and 63S BG (Bonding Chemical, Katy, TX) 2 and 4 wt %; these were labeled as MTABG2 and MTABG4, respectively. The composition of 63S BG was 63% SiO_2_, 31% CaO, and 6% P_2_O_5_, and its particle size was less than 20 μm. The concentrations were based on a previous study [11]. Liquid-to-powder (L/P) ratio of DW to the powder was 0.3 throughout the experiments, which was set according to the manufacturer’s recommendations. The samples were fabricated as discs to achieve a 10-mm diameter and 3-mm thickness and allowed to set for 24 h in a 5% CO_2_ incubator at 37 °C. The compositions of the materials used in this study are listed in Table 1. For each experiment, 6 samples of each experimental group were prepared and extracted using 12 ml of α-minimum essential medium (α-MEM; GIBCO, CA, USA) in a 5% CO_2_ incubator at 37 °C for 7 d followed by filtering through a syringe filter (PALL Life Sciences, USA) with 0.2-μm pore size. The eluates used in each experiment were freshly prepared before following biological experiments. The culture medium was replaced every 2 d, and DPSCs were subcultured on reaching confluence and used at passages 3 to 5.

**Table 1.** Materials used in this study

Group	Product name	Manufacturer	Chemical composition
MTA	ProRoot MTA	Dentsply Sirona, Tulsa, OK, USA	Powder: Tricalcium silicate (Ca_3_SiO_5_), dicalcium silicate (Ca_2_ SiO_4_), bismuth oxide (Bi_2_O_3_), tricalcium aluminate (Ca_3_Al2O_6_), calcium sulfate (CaSO_4_), gypsum (CaSO_4_·2H_2_O)
			Liquid: Distilled water (H_2_O)
BG	63S Bioglass	Bonding Chemical, USA	Silicon dioxide (SiO_2_), calcium oxide (CaO), phosphorus pentoxide (P_2_O_5_)

### Cell viability test

The viability of DPSCs from each experimental group was evaluated using cell counting kit-8 (CCK-8; Dojindo, Tokyo, Japan). In total, 2 × 10^4^ DPSCs/well were cultured on a 96-well plate (SPL Life Sciences, Korea) with growth media (GM) containing α-MEM with 10% fetal bovine serum and 1% penicillin/streptomycin. DPSCs in the experimental group with cement eluates were cultured for 24, 48, and 96 h. DPSCs cultured in intermediate restorative material (IRM; Dentsply Sirona, Tulsa, OK, USA) eluates served as the negative control group. For the CCK-8 assay, 1:10 diluted CCK-8 solution was rested for 4 h. Subsequently, 10 μl of the diluted solution was added to each 96-well plate well. Absorbance at 450 nm was measured using a SpectraMax enzyme-linked immunosorbent assay (ELISA) reader (Molecular Devices, Sunnyvale, CA, USA) [2,15].

### Cell migration and cell adhesion assay

A cell migration assay was conducted to evaluate DPSC migration ability. DPSCs (6 × 10^5^/well) were cultured in a scar block cell culture dish (SPL) and incubated in a culture medium for 24 h until a monolayer was formed. The upper insert was removed to create a 500-μm-wide cross-shaped wound on the cell culture dish. DPSCs in the experimental group with cement eluates were cultured for 24 h. DPSCs without cement eluates served as the control group. Images of the scratched areas at 0 and 24 h were captured using a microscope (LX-51; Olympus, Tokyo, Japan). The contraction area was calculated using ImageJ software (NIH, Bethesda, MD, USA) [15]. A cell adhesion assay was conducted to evaluate the collagen adhesion of DPSCs. Type I collagen-coated (Sigma-Aldrich, St Louis, MO, USA) 96-well plates (SPL) were washed with phosphate-buffered saline (PBS) to remove unbound collagen. A blocking buffer (1% bovine serum albumin in α-MEM, Sigma-Aldrich) was used to prevent nonspecific binding. Plates were washed with washing buffer, and 5 × 10^5^ DPSCs in 100 μl from the experimental group with cement eluates were added. DPSCs with no cement eluates served as the control group. Cells were incubated at 37 °C in a CO_2_ environment for 1 h. After washing out unbound cells, attached cells were fixed with 4% paraformaldehyde for 15 min. The cells were then washed and stained with crystal violet for 10 min. To evaluate the relative number of bound cells, 100 μl of 2% sodium dodecyl sulfate (SDS; Sigma-Aldrich) was added to extract crystal violet (Sigma-Aldrich), and absorbance at 570 nm was measured using a SpectraMax ELISA reader (Molecular Devices) [15].

### Preparation of immersion solution, SBF

Tris(hydroxymethyl)aminomethane [Tris; (CH_2_OH)_3_CNH_2_] SBF with pH 7.4 described by Tas [16] was used to investigate the effect of the storage solution on DPSC mineralization potential. The composition of the SBF is summarized in Table 2. The samples were set for 24 h and immersed in DW or SBF for 2 weeks. Subsequently, samples were prepared for scanning electron microscopy (SEM) analysis for surface morphology observation. For the mineralization assay, 6 samples were immersed in DW or SBF for 2 weeks and subsequently eluted in 6 ml of osteogenic media (OM) for 7 d, which contained α-MEM with 10% fetal bovine serum, gentamycin, dexamethasone (10 nM), l-ascorbic acid (100 μM), β-glycerophosphate (10 mM), and cement eluates. The prepared eluate was used for alkaline phosphatase (ALP) activity test, Alizarin red S (ARS) staining, Western blotting, and PCR.

**Table 2.** Simulated body fluid composition

Composition	Amount (g/l)
NaCl	6.547
NaHCO_3_	2.268
KCl	0.373
Na_2_HPO_4_·2H_2_O	0.178
MgCl_2_·6H_2_O	0.305
CaCl_2_·2H_2_O	0.368
Na_2_SO_4_	0.071
(CH_2_OH)_3_CNH_2_	6.057

### Morphology of MTA surface and DPSCs on the MTA

SEM was conducted to observe the cement surface, DPSC morphology, and their attachment to the cement surface. DW- and SBF-immersed samples were prepared. Two discs of MTA_DW and MTA_SBF groups were selected to observe the cement surface. One disc was placed in a well of a 12-well plate (SPL). Subsequently, 100 μl of GM with 1 × 10^5^ DPSCs were directly seeded onto the experimental group disc surfaces. After 4 h, 2 ml of GM was added, and cells were cultured for 3 d. The disks with DPSCs were fixed in 2.5% glutaraldehyde for 12 h, followed by dehydration in a graded series of ethanol concentrations up to 100% and immersion in 100% hexamethyldisilane for 30 min. Dried samples were sputter coated with gold/palladium (Polaron e5400 SEM Sputter Coating System; Bio-Rad, Kennett Square, PA, USA) before visualization [15]. SEM (Apreo S, Thermo Fisher Scientific, USA) images were captured at ×400, ×1,000, and ×2,500 magnifications.

### pH changes of MTA immersion solution

To investigate the ionic changes in the immersion solution, the pH measurement was performed using fresh mix samples. Each experimental sample was mixed and shaped into 3-mm-thick and 10-mm-diameter disks using rubber molds under aseptic conditions. The mixed samples were stored for 3 h at room temperature for the initial setting [17]. The set specimens were sterilized with ultraviolet light for 10 min on each surface. The disc was placed in a 10-ml test tube and treated with 2 immersion solutions: DW and SBF. The surface area-to-volume ratio used to prepare the eluate was approximately 250 mm^2^/ml, following the modified ISO standard 10993-5 [18]. One sample was used for pH measurement in each experimental group. Each test tube was sealed in a conical tube containing 5 ml of DW or SBF. The pH of each specimen was measured 3 times using an Aqua searcher AB33EC Bench Meter (Ohaus, NJ, USA) at 0 (baseline), 1, 2, 3, 4, 12, 24, and 48 h after shaking gently.

### Effect of BG-supplemented MTA on the osteo/odontogenic differentiation of DPSCs

For the ALP activity test, DW- and SBF-immersed samples were prepared and eluted in OM. To induce osteogenic differentiation, DPSCs were cultured in OM and MTA, and MTABG which were the 4 wt % BG incorporated with MTA cement. The negative control group was cultured in GM only, whereas the positive control group was cultured in OM only. Cells were incubated for 14 d, and their respective media were replaced every 2 to 3 d. The ALP activity of DPSCs was measured using an ALP assay kit (ab83369; Abcam, Cambridge, MA, USA) by the hydrolysis of p-nitrophenyl phosphate (50 μl) solution at 37 °C. After 1 h in the dark at 25 °C, the samples were then transferred, and the absorbance was measured at 405 nm using a SpectraMax ELISA reader (Molecular Devices). ALP activity was expressed as U/l protein [15].

For the ARS staining, DW- and SBF-immersed samples were prepared and eluted in OM. DPSCs were cultured in OM and cement eluates for 7, 14, and 21 d. Then, cells were washed with PBS, fixed in 70% ethanol, and stained with 300 μl of 2% ARS staining reagent (Sigma-Aldrich). After removing the staining reagent, the images were captured using a microscope (LX-51; Olympus, Tokyo, Japan). ImageJ software (NIH, Bethesda, MD, USA) was used to evaluate the areas of calcified nodules.

RNA extraction and real-time quantitative PCR (RT-qPCR) were also analyzed. DPSCs were seeded into 6-well plates and cultured in OM and cement extraction solution for 3 and 7 d. The cultured cells were then harvested, and total RNA was extracted using Trizol (Life Technologies). The RNA concentration was quantified using a Nanodrop ND 2000 spectrophotometer (Thermo Fisher Scientific, Waltham, MA, USA). For cDNA synthesis, 1 μg of total RNA was reverse transcribed using the First Strand cDNA Synthesis Kit (#K1612, Thermo Fisher Scientific). The resulting cDNA was subsequently amplified using Power SYBR Green PCR Master Mix (#4367659, Applied Biosystems) on a StepOnePlus Real-Time PCR System (Applied Biosystems, Life Technologies, Waltham, MA, USA). Primers for bone morphogenetic protein-2 (BMP-2), ALP, dentin matrix acidic phosphoprotein 1 (DMP-1), and dentin sialophosphoprotein (DSPP) were used for gene amplification. Glyceraldehyde-3-phosphate dehydrogenase (GAPDH) served as the housekeeping gene control. The primers were synthesized by Integrated DNA Technologies Inc. (IA, USA), and the gene expression was evaluated using the 2^−ΔΔCt^ method.

### Statistical analysis

Each experiment contained independent samples in triplicate and was repeated at least twice with qualitatively identical results. One-way analysis of variance and the Tukey post hoc test were used to determine the statistically significant differences in test materials. Differences were considered statistically significant at *P* < 0.05. All statistical analyses were performed using IBM SPSS statistics (version 29.0.1.0; IBM Corp., NY, USA).

## Results

**Fig. 1.**
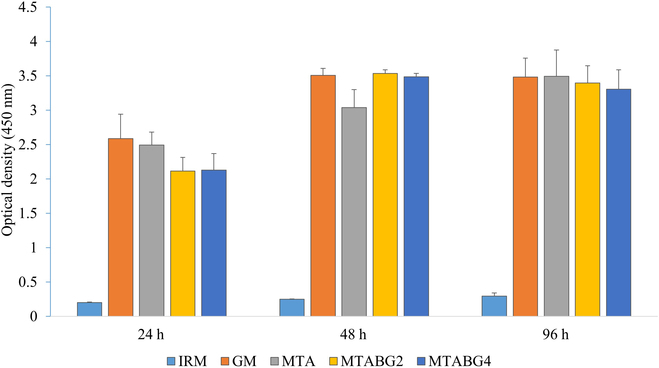
CCK-8 cell viability test results of human DPSCs in the MTA, MTABG2, and MTABG4 experimental groups for 24, 48, and 96 h. IRM was used as the negative control, while “no cement” was used as the positive control (GM). Statistically significant differences between experimental groups were indicated in different lowercase letters (*P* < 0.05).

The results of the cell viability test for the experimental groups are presented in Fig. 1. The IRM experimental group, serving as the negative control, exhibited significantly low cell viability (*P* < 0.05). For all experimental groups, cell viability was lower in the 24-h samples compared to the 48-h and 96-h samples (*P* < 0.05). There were no significant differences observed between BG concentrations in the experimental groups. In some experimental groups, cell viability was significantly lower than that of the positive control in the 24-h and 48-h samples (*P* < 0.05). However, no significant differences in cell viability were observed between the 96-h samples of the positive control group and the experimental groups.

**Fig. 2.**
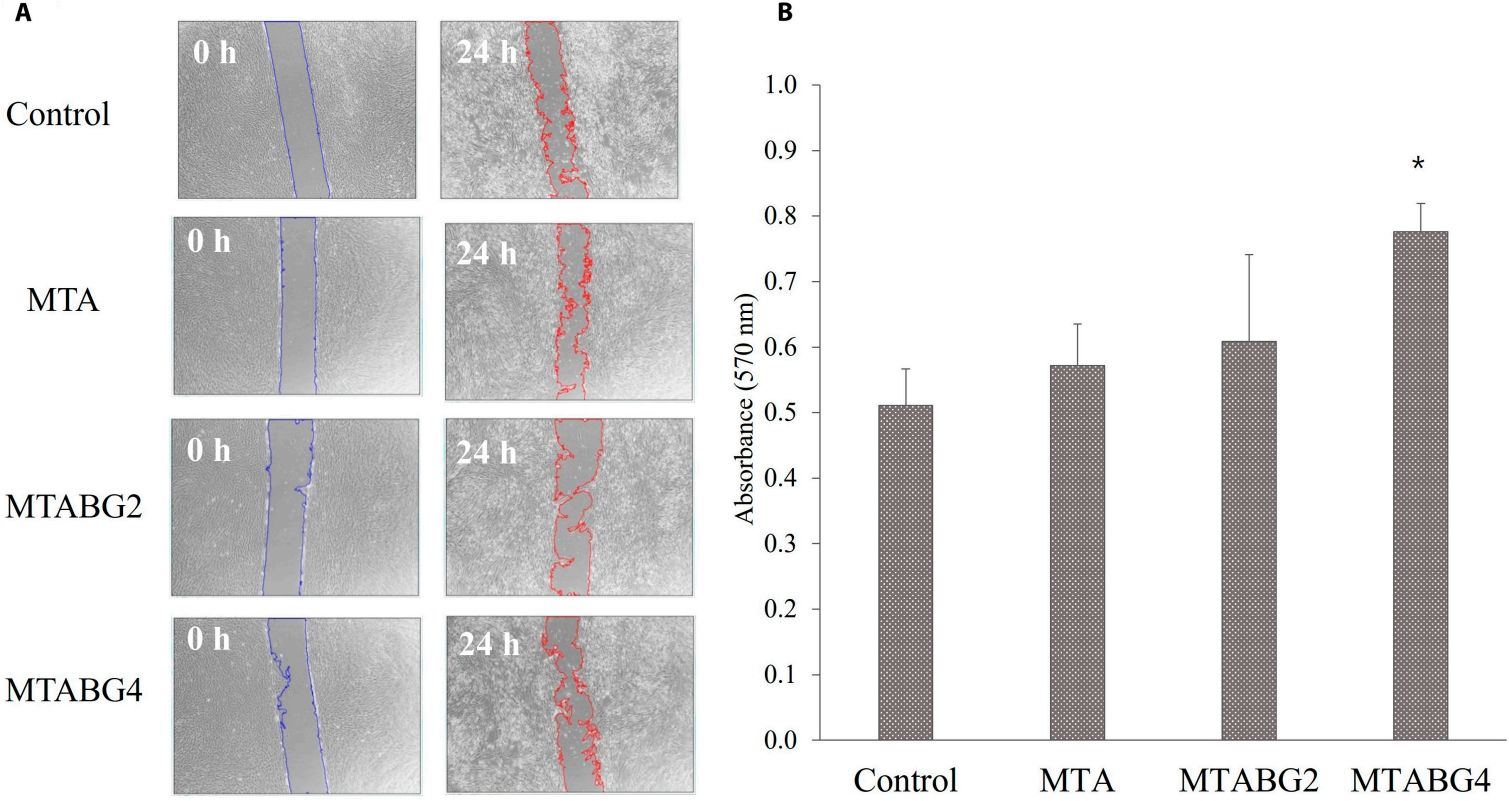
(A) Analysis of DPSCs by in vitro cell migration assay. There are no significant differences between experimental groups (*P* > 0.05). (B) Adhesion assay to type 1 collagen of human DPSCs by absorbance of crystal violet of each experimental group. Asterisk indicates a statistically significant difference between experimental groups (*P* < 0.05).

The results of the cell migration assay and adhesion assay are presented in Fig. 2 and Table 3. After 24 h, microscopic images showed cell migration toward the wounded site. The control group exhibited 26.79% wound contraction. The MTA, MTABG2, and MTABG4 groups showed 27.46%, 21.67%, and 22.53% wound contraction, respectively. The experimental groups exhibited a cell migration ability similar to that of the positive control group. No significant differences were observed between BG concentrations in the experimental groups (*P* > 0.05). For the adhesion assay, collagen-attached DPSCs were stained with crystal violet to quantify the number of stained cells. The MTABG4 group showed significantly higher cell adhesion (*P* < 0.05) than the other groups. No significant differences were observed between the MTA, MTABG2, and positive control group (*P* > 0.05).

**Table 3.** Cell migration assay and adhesion assay. Cell migration assay data and adhesion assay were presented as area (%), and no significant differences were observed between the experimental groups in the cell migration assay (*P* > 0.05). Adhesion to type I collagen of DPSCs by absorbance of crystal violet was shown in the right column, and MTABG4 showed the significant higher adhesion compared to other groups (*P* < 0.05).

Groups	Cell migration assay	Adhesion assay
Control	26.79 (3.44)	0.51 (0.06)
MTA	27.46 (10.77)	0.57 (0.06)
MTABG2	21.67 (6.47)	0.61 (0.13)
MTABG4	22.53 (2.78)	0.78 (0.04)*

* Statistical significance (*P* < 0.05)

**Fig. 3.**
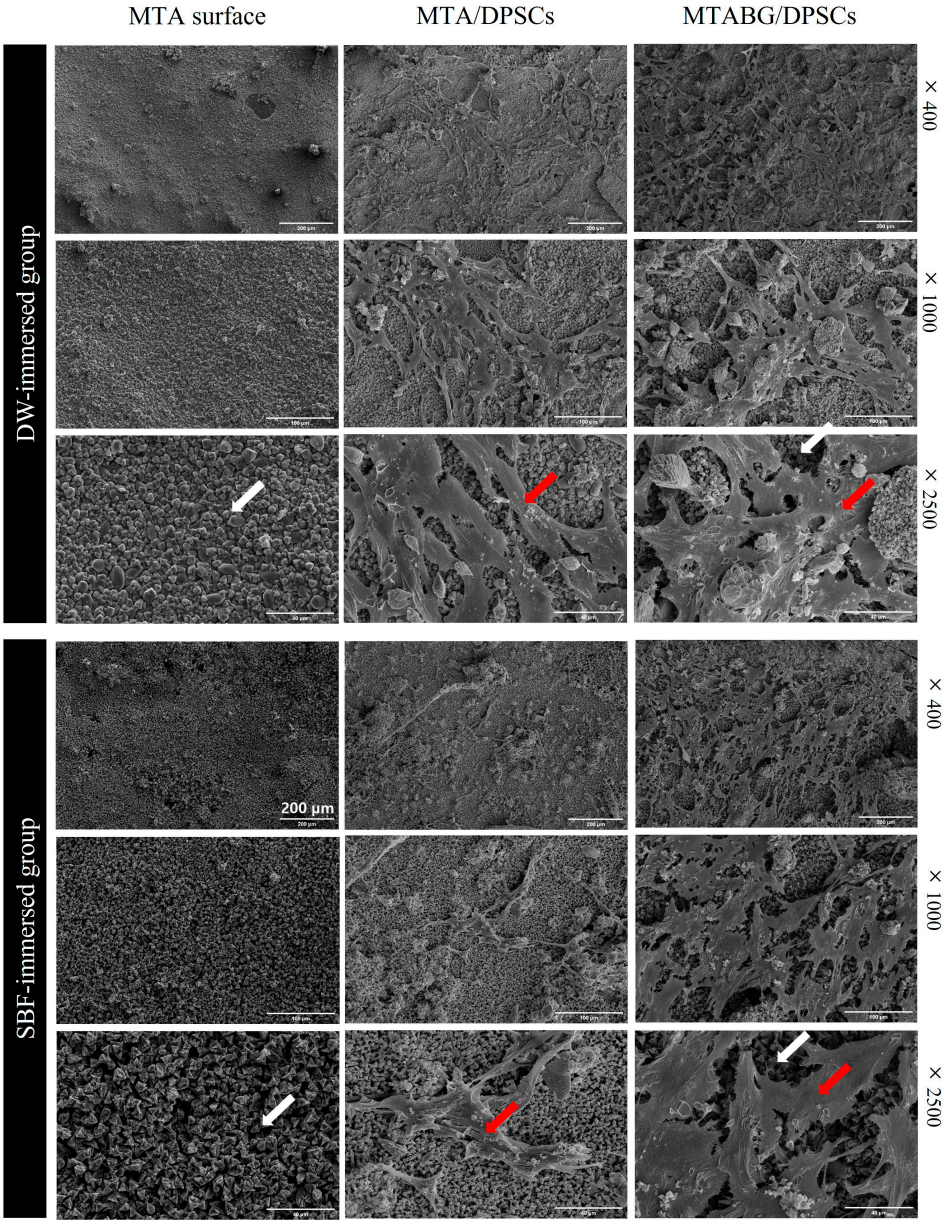
Representative SEM images of the MTA surface, and DPSCs on the experimental MTA surfaces: 2,500 MTA sample surfaces were commonly covered with mineralized crystals (white arrows). SEM images revealed that the DPSCs in each experimental group were able to attach to the surface material, showing the spindle-shaped fibroblastic features (red arrows).

The SEM images in Fig. 3 display the surfaces of MTA cement and the morphology of DPSCs on the disks from each experimental group after 3 d of culture. The MTA cement surface was covered with mineralized crystals, and DPSCs from each experimental group were able to attach to the surface and exhibited a spindle-shaped fibroblastic morphology.

**Fig. 4.**
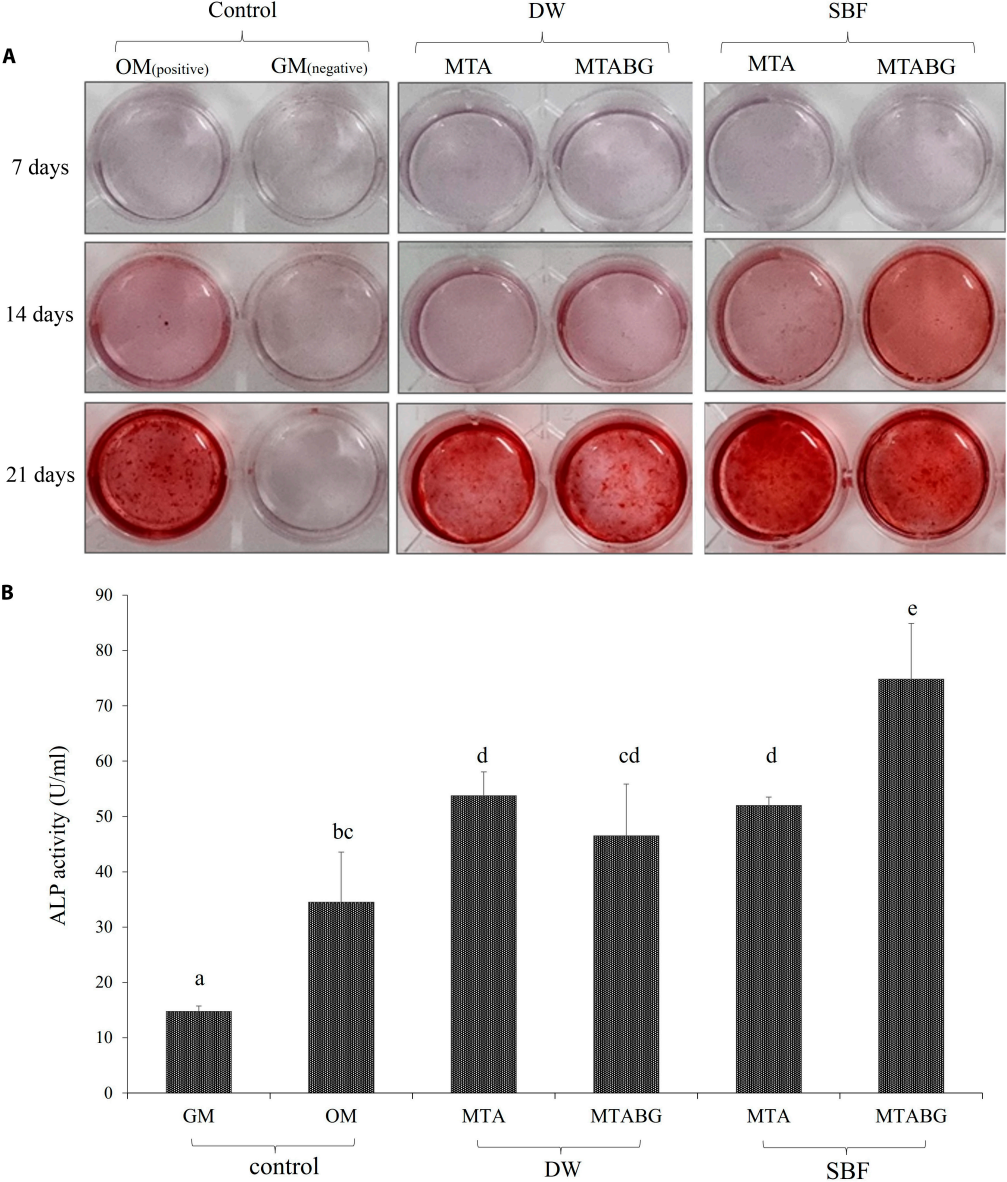
(A) ARS staining of DPSCs that were cultured for 7, 14, and 21 d. Images of DW- or SBF-treated MTA or MTABG specimens were eluated in the OM, and DPSCs were cultured. Osteogenic and growth medium served as positive and negative control group, respectively. Calcified nodules were stained with red color. (B) ALP activity test of experimental groups. Different lowercase letters represent statistically significant differences between time points within the same experimental group (*P* < 0.05).

The results of the pH measurements are presented in Table 4. In the DW groups, the baseline pH increased from 5.8 to approximately 12. In the SBF groups, the baseline pH increased from 7.4 to approximately 8 to 9. The pH changes slightly decreased over time, but the pH continued to increase for 48 h. The DW groups exhibited a higher pH increase than the SBF groups. No differences in pH were observed among the MTA, MTABG2, and MTABG4 experimental groups. Figure 4A shows the results of the ARS staining assay, indicating increased staining intensity over time. SBF-immersed groups displayed more intense staining than DW-immersed groups. On day 14, all experimental groups showed a positive correlation between the increase in BG concentration and the number of calcification nodules. All experimental groups had more calcified nodules than the negative control group.

**Table 4.** pH measurements of DW- and SBF-treated experimental groups. Data are presented as mean (SD). Each sample has measured the pH 3 times at each time point after calibration. pH was measured 3 h after mixing, achieved for the initial setting.

DW groups (pH: 5.8)	MTA	MTABG2	MTABG4
Immediate	12.26 (0.01)	12.47 (0.01)	12.25 (0.02)
1 h	11.98 (0.01)	12.11 (0.00)	12.19 (0.01)
2 h	12.21 (0.01)	12.34 (0.01)	12.36 (0.00)
3 h	12.15 (0.00)	12.18 (0.01)	12.28 (0.03)
4 h	12.26 (0.00)	12.18 (0.00)	12.26 (0.01)
12 h	12.18 (0.01)	12.08 (0.00)	12.31 (0.01)
24 h	11.75 (0.01)	11.82 (0.03)	12.22 (0.00)
48 h	11.97 (0.00)	11.84 (0.00)	12.23 (0.00)
SBF groups (pH: 7.4)	MTA	MTABG2	MTABG4
Immediate	8.68 (0.10)	9.48 (0.09)	8.44 (0.04)
1 h	10.96 (0.02)	8.82 (0.01)	8.73 (0.00)
2 h	9.02 (0.02)	9.65 (0.05)	8.39 (0.01)
3 h	8.06 (0.01)	8.29 (0.05)	8.08 (0.01)
4 h	8.10 (0.00)	8.31 (0.04)	7.95 (0.04)
12 h	7.88 (0.01)	8.22 (0.00)	7.99 (0.00)
24 h	8.17 (0.02)	8.42 (0.02)	7.86 (0.01)
48 h	7.93 (0.00)	8.03 (0.01)	7.90 (0.00)

The results of the ALP activity tests are shown in Fig. 4B. The negative and positive control groups showed activity levels of 14.74 and 34.48, respectively. In the DW-treated groups, there were no significant differences between MTA and MTABG. However, MTABG showed significantly higher ALP activity compared to MTA in the SBF-treated group (*P* < 0.01). All experimental groups exhibited significantly higher ALP activities than the control group (*P* < 0.05).

**Fig. 5.**
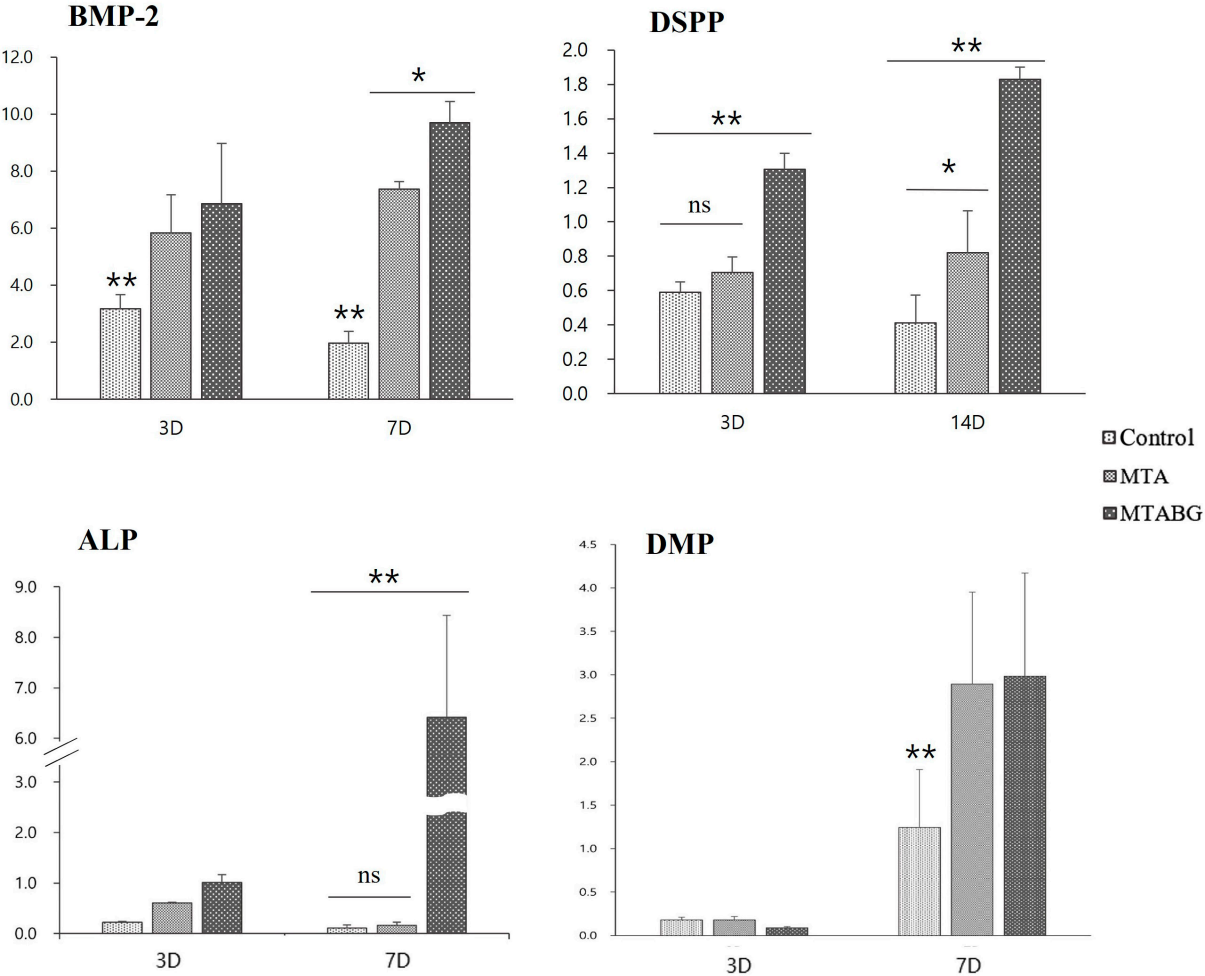
Osteogenic markers determined by real-time quantitative PCR (RT-qPCR).

The effect of MTA and BG supplementation on DPSCs was analyzed by real-time PCR to quantify the expression levels of marker genes, including DSPP, DMP-1, BMP-2, and ALP (Fig. 5). After 3 d of induction, BMP-2 mRNA levels of MTA and MTABG were significantly increased compared to the control group. DSPP showed a significant increase in the MTABG group compared to the control and MTA groups. There were no significant differences in the ALP and DMP-1. After 7 d of induction, MTA showed higher expression in BMP-2 and DSPP. On the other hand, MTABG exhibited significantly increased expression in all examined markers, including BMP-2, ALP, DSPP, and DMP-1.

## Discussion

Regenerative endodontics involves the use of dental materials like MTA and BG, which come into direct contact with body fluids or blood clots to facilitate pulp regeneration. These materials need to be nontoxic, compatible with the dentin–pulp complex, and capable of promoting dentin repair or mineralization. Previous studies have demonstrated the biocompatibility of MTA [3,4,8], while BG is known for its ability to interact with body fluids, releasing silica ions that promote mineralization [9]. Once BG contacts with body fluids or SBF, BG immediately undergo ionic dissolution and glass degradation via the exchange of H^+^ ions in the solution and Na^+^ and Ca^2+^ from the glass network. Then, these ionic exchanges lead the formation of silanol groups (Si–O–H) due to the hydrolysis of the silica, forming orthosilicic acid and Si(OH)_4_ on the surface in the form of a negatively charged gel. This unique gel layer functions as a matrix for hydroxyapatite with precipitation sites [19]. Our study focused on the biological properties of MTA combined with BG, which had previously shown improved dentin bond strength and compressive strength under specific storage conditions [11,14]. We also found that adding BG did not interfere with MTA’s setting process but increased the formation of hydroxyapatite during maturation [11].

We evaluated the impact of MTA and MTABG on the viability of DPSCs using the CCK-8 assay, which revealed increased cell viability over time. In the previous study [11], we examined the effect of adding BG on the physical properties of MTA and found that lower concentrations of BG, specifically 2 and/or 5 wt %, increased both the push-out bond strength and compressive strength under SBF storage conditions. Flores-Ledesma et al. [13] had also reported that adding more than 10 wt % of BG reduced the compressive strength of MTA. Based on the previous studies, we varied the concentration of BG supplementation into MTA less than 5% and investigate the biological behavior of DPSCs. The 24-h groups showed lower cell viability than the 48-h and 96-h groups. By the 96-h mark, there were no significant differences between experimental groups, indicating that MTA and BG supplementation had no detrimental effect on DPSC viability (Fig. 1). Additionally, we observed that the experimental groups had lower cell viability than the positive control group after 24 h, likely due to the highly alkaline environment created by MTA and BG, which decreased in harmful effects over time. This is consistent with previous studies indicating a decrease in cell viability initially, followed by recovery over time [15,18]. Another study [20] reported that MTA extracts had slightly higher cell viability at 48 h and slightly lower cell viability at 72 h compared to the control group. The 1:4 dilution of cement extract affected the cell viability of DPSCs in our study.

Cell migration and cell adhesion are essential processes for tissue repair. In this study, the cell migration assay revealed no significant differences between the positive control and experimental groups (Fig. 2A). In the previous study, we investigated that MTA and supplementation of BG induced to formulate the hydroxyapatite-like crystals [11] and hydroxyapatite had been reported to stimulate the cellular adhesion by fibrinogen attachment [21]. Furthermore, the MTA extract-treated cells show accelerated stress fiber assembly and a distinct vinculin expression, which is essential for focal adhesion [22]. Thus, MTA and BG supplementation did not alter the cell migration of DPSCs. The cell adhesion assay revealed that the MTABG4 group had high cell adhesion, while the other experimental groups demonstrated a cell adhesion similar to that of the control group (Fig. 2B). It is speculated that the more significant release of calcium ions from BG might have affected the cell adhesion ability of DPSCs to collagen.

We immersed the sample in DW or SBF for 2 weeks for hydroxyapatite formation. MTA and BG have a high concentration of calcium ions at the interface and form hydroxyapatite with phosphate ions from SBF [23,24]. Chen et al. [25] reported that SBF circulation to demineralized collagen in dentine did not induce significant remineralization but generated needle-like crystals. SBF also has calcium ions; thus, we compared the SBF-treated group with the DW-treated group without ionic components. SEM images showed the adhesion of DPSCs to the cement surface in all experimental groups (Fig. 3). Both cement surfaces immersed in DW and SBF were covered with mineralized crystal, and DPSC proliferation was observed on the cement surface, which showed biocompatibility on DPSCs, and it was coincident with that described in our previous study [11]. We found that the addition of BG into MTA exhibited precipitates in the dentinal tubules, as observed through SEM. Additionally, x-ray diffraction (XRD) analysis revealed that these precipitates were associated with increased hydroxyapatite formation.

When MTA powder is mixed with DW, calcium hydroxide and calcium silicate hydrate are initially formed, eventually creating a calcium precipitate [17]. This precipitated calcium then produces calcium hydroxide, which is the cause of the high alkalinity of MTA after hydration. DW- and SBF-treated groups showed an increase in pH continuously observed for up to 48 h, even after replacing the solution, indicating the ongoing production of calcium hydroxide. A low pH was observed in the SBF group because of the buffering capacity of SBF. We used Tris to prepare the SBF (Table 2). Tris buffer is widely utilized in biological research due to its p*K*_a_ 8.07 at room temperature. The p*K*_a_ can vary slightly different with temperature, because the dissociation constant (*K*_a_) is temperature-dependent. It adjusted with hydrochloric acid, and it provides an effective buffering range from 7.07 to 9.07, making it ideal for dissolution experiments that replicate physiological pH conditions [24]. Cerruti et al. [26] compared BG immersion in Tris buffer with immersion in DW and reported that the ion release in the Tris buffer solution of pH 7.4 and 6.9 solutions significantly surpassed that in DW. Those BGs immersed in Tris-buffered SBF solution showed superior calcium ion release, likely owing to the rapid formation of Ca-containing salts that are less soluble at high pH values.

Based on these results, we examined the effects of odontoblastic differentiation and mineralization on the experimental groups via the ALP activity test, ARS staining, and PCR analysis of experimental groups with 2 DW- and SBF-treated variables. Bakopoulou et al. [27] reported that ALP exhibited a prominent expression after the osteogenic differentiation of DPSCs, concluding that ALP is an early osteogenic/odontogenic differentiation biomarker. In regenerative endodontic procedures, ALP releases free phosphate ions. Phosphate ions react with calcium ions to form a calcium phosphate precipitate and hydroxyapatite. In this study, the SBF-treated MTABG group showed the highest ALP activity among all groups, and all experimental groups showed significantly higher ALP activity than the control group. ARS staining was used to evaluate the mineralization of the extracellular matrix in each experimental group. Higher staining with osteogenic differentiation was observed from 14 d in the SBF-treated group, particularly for the BG supplementation groups. Calcium and silicon ions are known to stimulate osteogenic differentiation [28]. BG supplementation influences osteogenic differentiation by releasing more silica ions, which can induce the precipitation of hydroxyapatite [29].

The effect of MTA and BG supplementation into MTA to DPSCs was analyzed by real-time PCR to quantify the expression levels of marker genes, which includes DSPP, DMP-1, BMP-2, and ALP (Fig. 5). Noncollagenous proteins such as BMP-2, DSPP, DMP-1, and ALP have been widely investigated markers that indicate the odontoblast or osteogenic differentiation markers. Our study showed increased BMP-2 mRNA levels in the MTA and MTABG after 3 d of induction, and MTABG showed significantly higher BMP-2 expression after 7 d of induction. BMPs are structurally related to the transforming growth factor β superfamily, which plays important roles during odontogenic differentiation [30]. BMP-2 is initially expressed in the dental epithelial cells at the embryonic stage and then shifts to the dental mesenchymal papilla and is involved in specifying the fate of the dental mesenchymal cells at a later stage of tooth development [31]. In this study, DSPP also showed a significant increase in the MTABG group compared to the control and MTA group after 3-d and 7-d induction, respectively. DSPP, as a differentiation marker of odontoblasts, is regulated by BMP-2. Yang et al. recently highlighted a novel signaling pathway in which BMP-2 activates DSPP gene after 3-d induction. MTABG showed significantly higher expression of ALP, and MTA and MTABG exhibited an increase in DMP-1. ALP and DMP are the most popular markers that indicate the early marker of odontoblastic differentiation. DMP is known to be related to the matrix mineralization of bone and dentine [32,33].

In this study, we used the primary cultured DPSCs isolated from supernumerary teeth with donors ranging in age from 6 to 20 years, and those DPSCs were pooled together. We used the primary cultured DPSCs, passage 3 to 5 cells. There are 3 main types of stem cells investigated for potential therapeutic applications in medicine: embryonic stem cells, induced pluripotent stem cells, and adult stem cells (ASCs) [34]. The clinical use of embryonic and induced pluripotent stem cells is limited due to significant drawbacks, including the risks of immune rejection, teratoma formation, and stringent ethical regulations [35]. Consequently, ASCs, including orofacial bone marrow stem cells, stem cells from exfoliated deciduous teeth, periodontal ligament stem cells, dental follicle stem cells, and DPSCs used in this study, are frequently used in regenerative medicine. Despite their utility, there are still concerns regarding the accessibility and variability in the quantity and characterization of isolated stem cells from different individuals, as well as their immunomodulatory properties [36,37]. Also, cultured primary stem cells do not grow infinitely but undergo only a limited number of cell division, which is known as cellular senescence. Recent studies had reported that human mesenchymal stem cells (MSCs) responded to injury and caused functional alterations by activation of stress-induced premature senescence such as radiation, heat shock, and chemotherapeutic materials [37–40]. To overcome these limitations caused by the use of DPSCs, more in vitro experiments with DPSCs originated from various spectrum of donor age and inflammation of origin tissue and preclinical animal in vivo studies should be required for translation in endodontic regeneration. Also, further clinical studies with new MTABG pulp-capping materials, including diverse populations and varying degrees of pulp inflammation, are required.

Our study result showed that BG supplementation of MTA exhibited biocompatibility comparable to that of conventional MTA, which was examined by CCK-8, cell migration, and cell adhesion assays, as well as cell morphology on cement surfaces. Under SBF treatment, BG-supplemented MTA presented a higher mineralization potential, particularly at a concentration of 4 wt % for the ALP activity assay, than the conventional MTA. More dense ARS staining and higher ALP activity, and higher expression of DSPP, ALP, and BMP-2 were shown in the SBF-treated MTABG. Within the limitations of this study, it could be concluded that BG supplementation might enhance the mineralization potential of MTA under biomimicking SBF treatment conditions.

## Data Availability

The datasets during and/or analyzed during the current study are available from the corresponding author on reasonable request.
